# High prevalence of associated injuries in anterior cruciate ligament tears: A detailed magnetic resonance imaging analysis of 254 patients

**DOI:** 10.1007/s00256-024-04665-9

**Published:** 2024-03-27

**Authors:** Riccardo Cristiani, Fabian van de Bunt, Joanna Kvist, Anders Stålman

**Affiliations:** 1 Department of Molecular Medicine and Surgery, Stockholm Sports Trauma Research Center, Karolinska Institutet, Stockholm, Sweden; 2 Capio Artro Clinic, FIFA Medical Centre of Excellence, Sophiahemmet Hospital, Valhallavägen 91, 11486 Stockholm, Sweden; 3 Division of Radiology, Skåne University Hospital, Skåne, Sweden; 4 Department of Health, Medicine and Caring Sciences, Division of Prevention, Rehabilitation and Community Medicine, Unit of Physiotherapy, Linköping University, Linköping, Sweden; 5 Center for Medical Image Science and Visualization (CMIV), Department of Health, Medicine and Caring Sciences, Linköping University, Linköping, Sweden

**Keywords:** Anterior cruciate ligament, ACL, Meniscus, Associated injuries, Magnetic resonance imaging, MRI

## Abstract

**Objectives:**

To evaluate the type and prevalence of associated injuries by using magnetic resonance imaging (MRI) in patients with anterior cruciate ligament (ACL) tears.

**Methods:**

Data from the Natural Corollaries and Recovery after ACL injury multicenter longitudinal cohort study were analyzed. Between May 2016 and October 2018, patients aged between 15 and 40 years, who had experienced an ACL tear within the last 6 weeks and sought medical attention at one of seven healthcare clinics in Sweden, were invited to participate. The mean time from injury to MRI was 19.6 ± 15.2 days. An orthopedic knee surgeon and a musculoskeletal radiologist reviewed all the MRI scans. The following structures were assessed: posterior cruciate ligament (PCL), medial collateral ligament (MCL) complex, lateral collateral ligament (LCL), popliteus tendon, medial meniscus (MM), lateral meniscus (LM), and cartilage. In addition, the presence of bone bruising, impaction fractures in the lateral femoral condyle (LFC) or posterolateral tibia (PLT), and Segond fractures were also assessed.

**Results:**

A total of 254 patients (48.4% males) with a mean age of 25.4 ± 7.1 years were included. The prevalence of associated injuries was as follows: PCL (0.4%), MCL {41.3% [superficial MCL and deep MCL (dMCL) 16.5%; isolated dMCL 24.8%]}, LCL (2.4%), MM (57.4%), LM (25.2%), cartilage (15.0%), bone bruising (92.9%), impaction fracture in the LFC (45.7%) and PLT (4.7%), and Segond fracture (7.5%).

**Conclusions:**

The prevalence of associated injuries in patients with ACL tears was high. The findings reported in this study may serve as a reference tool for orthopedic surgeons and radiologists in the diagnosis of associated injuries using MRI in patients with ACL tears.

## Introduction

Anterior cruciate ligament (ACL) tears are rarely isolated [1–4]. Thus, recognition of associated injuries in patients with ACL tears is essential. Misdiagnosed or untreated peripheral laxities are known causes of ACL graft failure [5]. LaPrade et al. [6] demonstrated that ACL graft forces increased significantly with varus loading at 0 and 30 degrees of flexion and internal rotation, after sectioning the lateral collateral ligament (LCL). Deficiency of the medial collateral ligament (MCL) complex is responsible for significantly increased forces in the ACL during valgus and external rotation [7, 8]. Meniscal and cartilage injuries are frequently observed in patients with ACL tears [1, 3, 9], and they are associated with increased knee laxity [3, 10], inferior subjective knee outcomes, and osteoarthritis development [11–13]. Meniscal ramp lesions, which can be easily overlooked during standard arthroscopic evaluation (using the anteromedial and anterolateral portals) [14, 15], have been associated with increased anterior tibial translation, internal and external rotation, pivot shift [16, 17], and accelerated cartilage degeneration in the medial compartment [18]. Bone bruising patterns are regarded as a footprint of the mechanism of injury, offering insights into possible associated injuries [19–22]. Finally, impaction fractures of the lateral femoral condyle (LFC), posterolateral tibia (PLT), and the Segond fracture have been associated with poorer outcomes and increased anterolateral rotatory laxity in ACL-injured knees [23–25].

Therefore, knowledge about the presence of associated injuries in ACL tears is essential for the orthopedic surgeon to maximize the treatment outcome of patients with ACL injuries.

Magnetic resonance imaging (MRI) is regarded (due to its high accuracy in evaluating soft tissues) as the best imaging modality to diagnose associated injuries in ACL tears [26].

The purpose of the present study was to provide a comprehensive analysis, which can be used as a reference tool for radiologists and orthopedic surgeons, regarding the type and prevalence of associated injuries on MRI in patients with ACL tears.

## Materials and methods

Ethical approval was obtained from the Regional Ethics Committee of Linköping, Sweden (no. 2016/44–31 and 2017/221–32). All patients provided written informed consent before participation. Data were extracted from the Natural Corollaries and Recovery after ACL injury (NACOX) study [27]. Patients were recruited between May 2016 and October 2018 from seven orthopedic clinics across Sweden. The inclusion criteria were an ACL injury sustained no more than 6 weeks before presentation and an age between 15 and 40 years at the time of injury. The exclusion criteria were previous ACL injury or ACL reconstruction (ACLR), fractures requiring separate treatment, inability to understand written or spoken Swedish language, cognitive impairments, or other illnesses or injuries that impaired function (e.g., fibromyalgia, rheumatic diseases, or other diagnoses associated with chronic pain). ACL tears were clinically diagnosed by an orthopedic surgeon and verified using MRI. In this study, only patients with available MRI data were included. Patients who only had a clinical diagnosis of ACL injury were not included.

The MRI prevalence and the factors associated with meniscal ramp lesions and MCL complex injuries from the NACOX study have been previously reported [1, 2].

### Radiological assessment

A total of 210 patients underwent MRI at two institutions (Capio Artro Clinic, Stockholm Sweden, and Linköping University Hospital, Linköping, Sweden). The remaining patients participating in the NACOX study (n = 44) underwent MRI at other institutions [27]. MRI was performed using a 1.5-T (Siemens) (n = 115) and 3.0-T scanners (Philips) (n = 139). A detailed description of MRI sequences is reported in Table 1. A sensitivity analysis of the MRI scanners (1.5-T vs. 3.0-T) is reported in the Appendix Table 4. The mean time from injury to MRI was 19.6 ± 15.2 days. All MRI examinations were retrospectively and independently assessed by an orthopedic knee surgeon (R.C.) and a musculoskeletal radiologist (F.v.d.B.) (fair interrater reliability: median Kappa 0.51). A conjoined assessment was performed to reach a consensus in the event of inconsistencies.

**Table 1:** Description of MRI sequences

3.0 T Scan
Sagittal PD TSE, 3 mm slice thickness with 0.3 mm gap. TE = 20 ms; TR = 1800 ms; ETL 10; FOV 160 × 145; Scan time 2:58 min Axial PD FatSat TSE, 3 mm slice thickness with 0.3 mm gap. TE = 35 ms; TR = 3981 ms; ETL 15; FOV 140 × 140; Scan time 4:15 min Sagittal PD FatSat TSE, 3 mm slice thickness with 0.3 mm gap. TE = 30 ms; TR = 3400 ms; ETL 15; FOV 160 × 145; Scan time 3:56 min Coronal PD FatSat TSE, 3 mm slice thickness with 0.3 mm gap. TE = 30 ms; TR = 3572 ms; ETL 16; FOV 160 × 140; Scan time 3:56 min
1.5 T Scan
Sagittal T1 TSE, 3 mm slice thickness with 0.5 mm gap. TE = 9.4 ms; TR = 450 ms; ETL 3; FOV 160 × 160; Scan time 2:24 min Axial PD FatSat TSE, 4 mm slice thickness with 1 mm gap. TE = 47.0 ms; TR = 3500 ms; ETL 15; FOV 160 × 160; Scan time 1:50 min Sagittal PD FatSat TSE, 3 mm slice thickness with 0.5 mm gap. TE = 56 ms; TR = 2720 ms; ETL 8; FOV 160 × 160; Scan time 2:28 min Coronal PD FatSat TSE, 3 mm slice thickness with 0.5 mm gap. TE = 56.0; TR = 2550 ms; ETL 8; FOV 160 × 160; Scan time 1:59 min

*ETL* echo train length, *FOV* field of view, *MRI* magnetic resonance imaging, *PD* proton density, *TE* echo time, *TR* repetition time, *TSE* turbo spin echo

The classification of associated injuries was based on the ACL Osteoarthritis Score (ACLOAS) for ligament, meniscus, and cartilage injuries [28]; Greif classification for meniscal ramp lesions [29]; and Sanders classification for bone bruising patterns [19]. The classification and grading were as follows:

- The posterior cruciate ligament (PCL) was classified as intact or injured.
- The MCL and LCL were classified as intact, partially or completely torn. Partial tears were defined as a partial rupture or discontinuity with preserved fibers, whereas complete tears were defined as complete ligament disruption [28]. Injury localization was also assessed [proximal (proximal third), mid-substance (central third), or distal (distal third)] [28]. A deep MCL (dMCL) injury was defined as a tear of the meniscofemoral and/or meniscotibial ligaments. Isolated dMCL tears were defined as isolated tears of the meniscofemoral and/or meniscotibial ligaments with intact superficial MCL (sMCL) [2]. The posterior oblique ligament (POL) was classified as intact or injured. Similarly, the popliteus tendon was classified as intact or injured.
- The medial meniscus (MM) and lateral meniscus (LM) were classified as normal, horizontal tears, radial and vertical tears, bucket handle tears, complex tears, root tears, and ramp lesions (for the MM) [28]. The locations were registered as posterior horn, corpus, or anterior horn. MM ramp lesions were classified into seven subtypes according to Greif et al. [29]: type 1, meniscocapsular ligament tears; type 2, partial superior peripheral meniscal horn tears; type 3A, partial inferior peripheral posterior horn meniscal tears; type 3B, meniscotibial ligament tears; type 4A, complete peripheral posterior horn meniscal tears; type 4B, complete meniscocapsular junction tears; and type 5, peripheral posterior horn meniscal double tears.
- Cartilage lesions were classified as partial thickness, full thickness, or degeneration [28]. The injury locations were the medial femoral condyle (MFC), lateral femoral condyle (LFC), medial or lateral tibial plateau, trochlea, or patella.
- Bone bruising patterns were documented and classified as described by Sanders et al. [19] in pivot-shift, dashboard, and hyperextension injuries. Pivot-shift bone bruising was defined as the presence of bone marrow edema in the posterolateral area of the lateral tibial plateau and the central area of the LFC. If the bone bruising pattern did not fit any of the patterns described by Sanders et al. [19], it was classified as “other”. In addition, bone bruising in the MFC and posteromedial tibia (PMT) was also assessed.
- Impaction fractures in the LFC and posterolateral tibia (PLT) were defined as depressions with a normal or injured cartilage surface [28].
- The presence of a Segond fracture was also documented [30].

### Statistical analysis

The present study serves as a descriptive study. All data were analyzed using Microsoft Excel (version 2018).

## Results

A total of 275 patients were included in the NACOX study. Eight patients only had a clinical diagnosis of ACL injury (no MRI), and the MRI scans of 13 patients were not available for analysis. Finally, a total of 254 patients (48.4% males) with a mean age of 25.4 ± 7.1 years and a mean body mass index of 23.8 ± 3.3 were included. The median (range) pre-injury Tegner activity score was 7 (2–9). Activity at the time of injury was as follows: football (37.4%), skiing (18.5%), floorball (13.4%), handball (5.9%), daily life activities (3.1%), martial arts (2.4%), basketball (2.4%), and others (16.7%).

Only one patient (0.4%) was identified with a PCL tear.

### MCL complex and LCL injuries

**Fig. 1:**
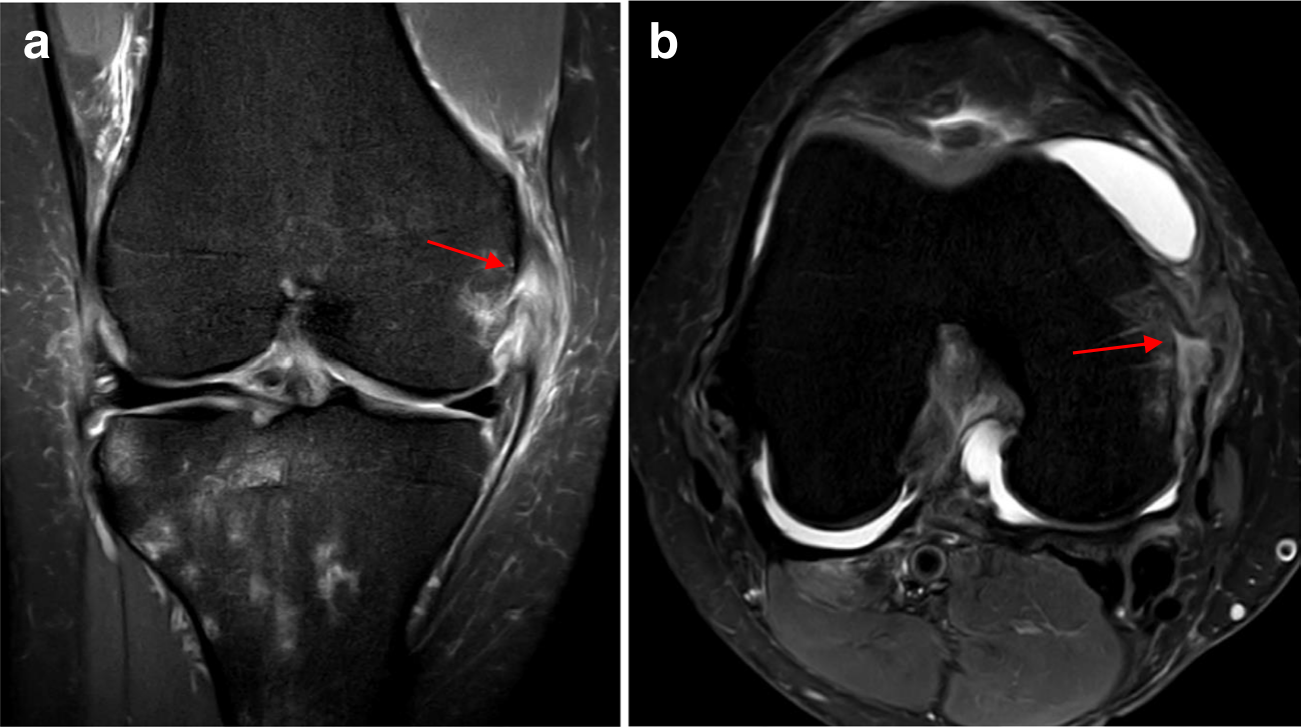
Complete proximal medial collateral ligament tear (superficial and deep) (arrows) on coronal (**a**) and axial (**b**) proton density fat saturation magnetic resonance imaging sequences (right knee)

**Fig. 2:**
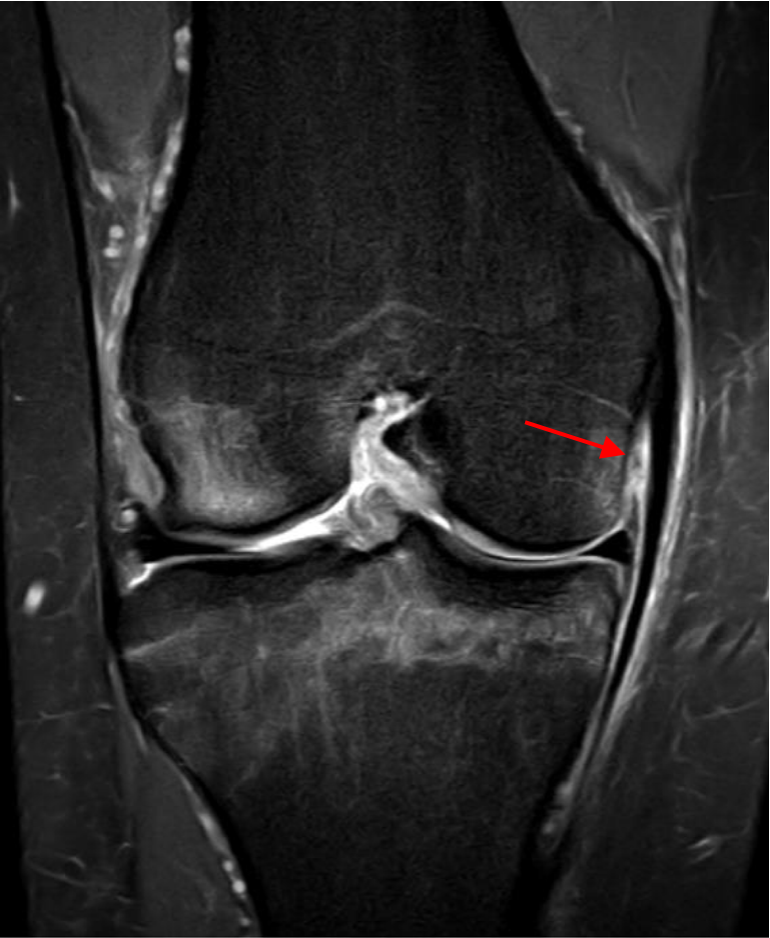
Isolated deep medial collateral ligament tear (meniscofemoral ligament) (arrow) on coronal proton density fat saturation magnetic resonance imaging sequence (right knee)

**Fig. 3:**
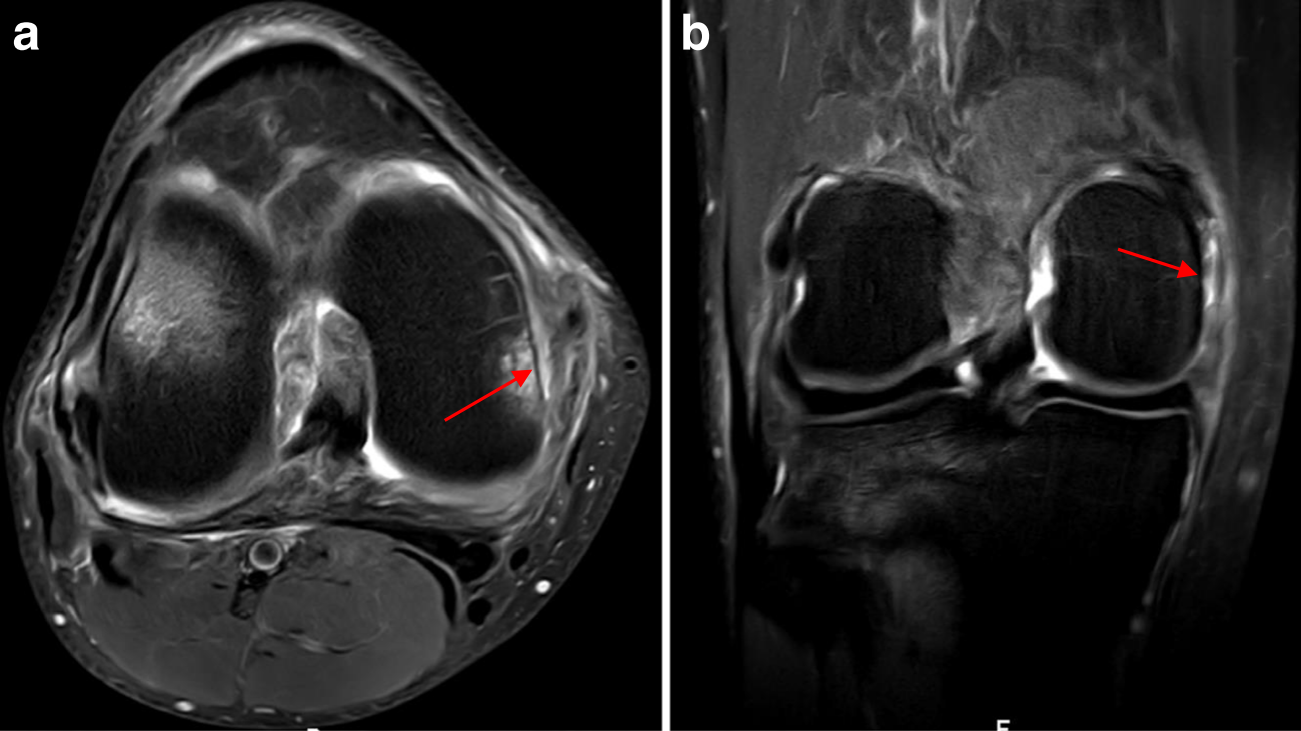
Posterior oblique ligament injury (arrows) on axial sequence above level of the joint line (**a**) and on coronal sequence posterior to the medial collateral ligament (**b**) on magnetic resonance imaging sequences (right knee)

Overall, MCL (sMCL and dMCL) injuries (Fig. 1) were identified in 42 patients (16.5%). Isolated sMCL injuries were not observed. In the event of an sMCL injury, a dMCL (particularly the meniscofemoral ligament) injury was always present. The severity and location of the sMCL injuries are displayed in Table 2. Isolated dMCL injuries (Fig. 2) were observed in 63 patients (24.8%). All but one tear involved the meniscofemoral ligament. Only one patient had an isolated tear of the meniscotibial ligament. POL injuries (Fig. 3) were observed in 12 patients (4.7%). The POL was never injured in isolation. In all the cases, an MCL (sMCL and dMCL) injury was present [2].

**Table 2:** Injury severity and location of sMCL and LCL injuries

	sMCL	LCL
Injury severity		
Partial	29 (69.0)	6 (100)
Complete	13 (31.0)	
Injury location		
Proximal	32 (76.2)	4 (66.7)
Mid-substance	2 (4.8)	2 (33.3)
Distal	8 (20)	

Data are reported as n (percentage)

*sMCL* superficial medial collateral ligament, *LCL* lateral collateral ligament

**Fig. 4:**
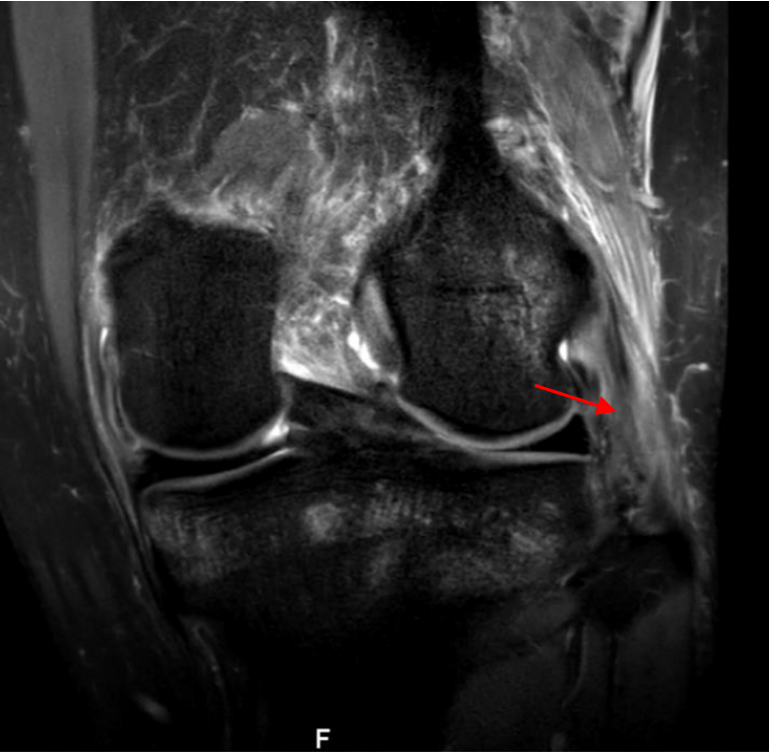
Partial mid-substance lateral collateral ligament tear (arrow) on coronal proton density fat saturation magnetic resonance imaging sequence (left knee)

Lateral collateral ligament injuries (Fig. 4) were identified in six patients (2.4%). The severity and location of the injuries are reported in Table 2. No popliteal tendon injuries were observed.

### MM and LM injuries

**Fig. 5:**
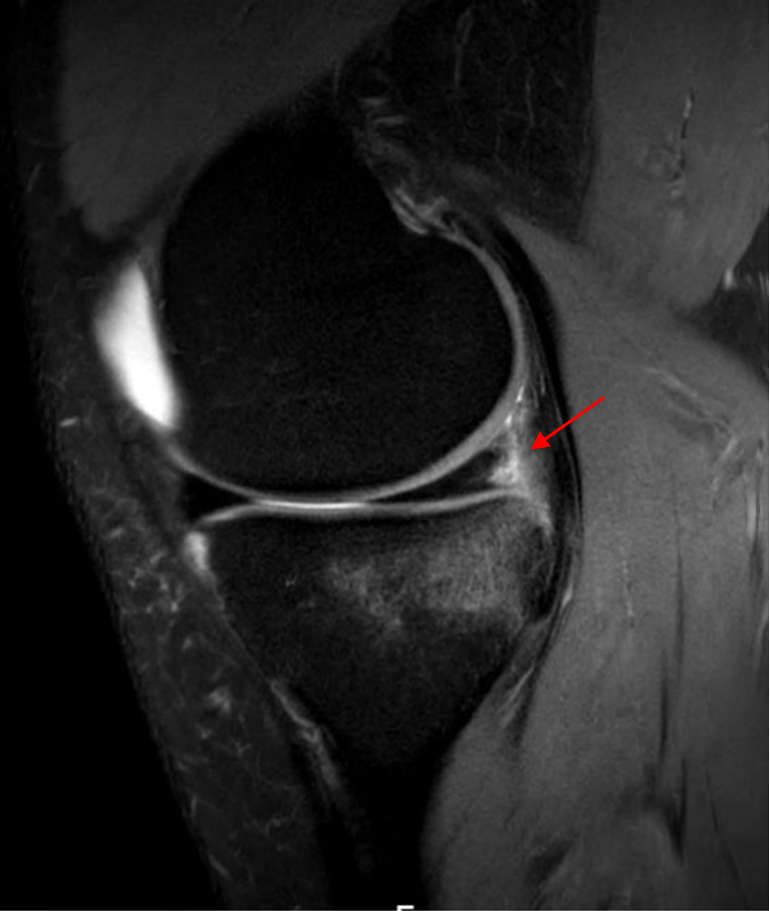
Meniscal ramp lesion type 4B (complete meniscocapsular junction tear) (arrow) has shown by the fluid intensity signal extending from the superior to the inferior articular surface on sagittal proton density fat saturation magnetic resonance imaging sequence (right knee)

**Fig. 6:**
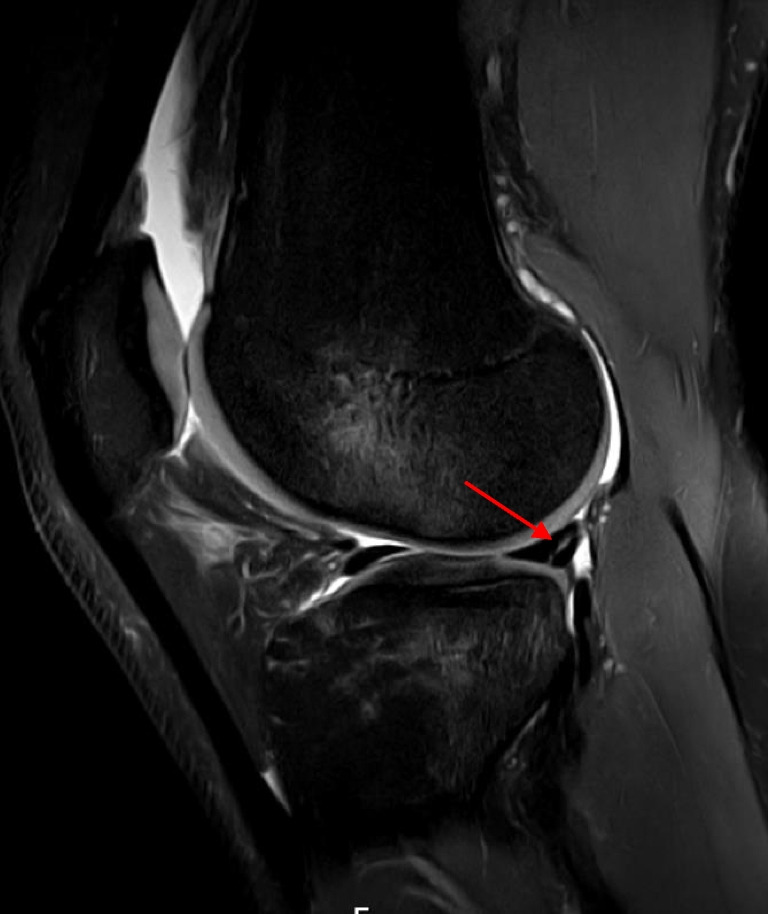
Radial-vertical lateral meniscus posterior horn tear (arrow) on sagittal proton density fat saturation magnetic resonance imaging sequence (right knee)

Overall, MM and LM injuries were identified in 146 (57.4%) and 64 (25.2%) patients, respectively. The types, distributions, and locations of injuries are reported in Table 3. Figure 5 displays a meniscal ramp lesion type 4B (complete meniscocapsular junction tear), whereas Fig. 6 demonstrates a radial-vertical LM posterior horn tear.

**Table 3:** Type and location of MM and LM injuries

	MM	LM
Injury type		
Horizontal	5 (3.4)	1 (1.6)
Radial and vertical	19 (13.0)	48 (75.0)
Bucket-handle	5 (3.4)	4 (6.2)
Complex	15 (10.3)	6 (9.4)
Root	2 (1.4)	5 (7.8)
Ramp lesions	100 (68.5)	
- Type 1, meniscocapsular ligament tear	13 (13.0)	
- Type 2, partial superior peripheral meniscal horn tear	4 (4.0)	
- Type 3A, partial inferior peripheral posterior horn meniscal tear	7 (7.0)	
- Type 3B, meniscotibial ligament tear	7 (7.0)	
- Type 4A, complete peripheral posterior horn meniscal tear	20 (20.0)	
- Type 4B, complete meniscocapsular junction tear	43 (43.0)	
- Type 5, peripheral posterior horn meniscal double tear	6 (6.0)	
Injury location		
Posterior horn	39 (26.7)	40 (62.5)
Corpus		15 (23.5)
Anterior horn		
Other (bucket-handle tear, root tear, ramp lesions for the MM)	107 (73.3)	9 (14.0)

Data are reported as *n* (percentage)

*MM* medial meniscus, *LM* lateral meniscus

### Cartilage injuries

**Fig. 7:**
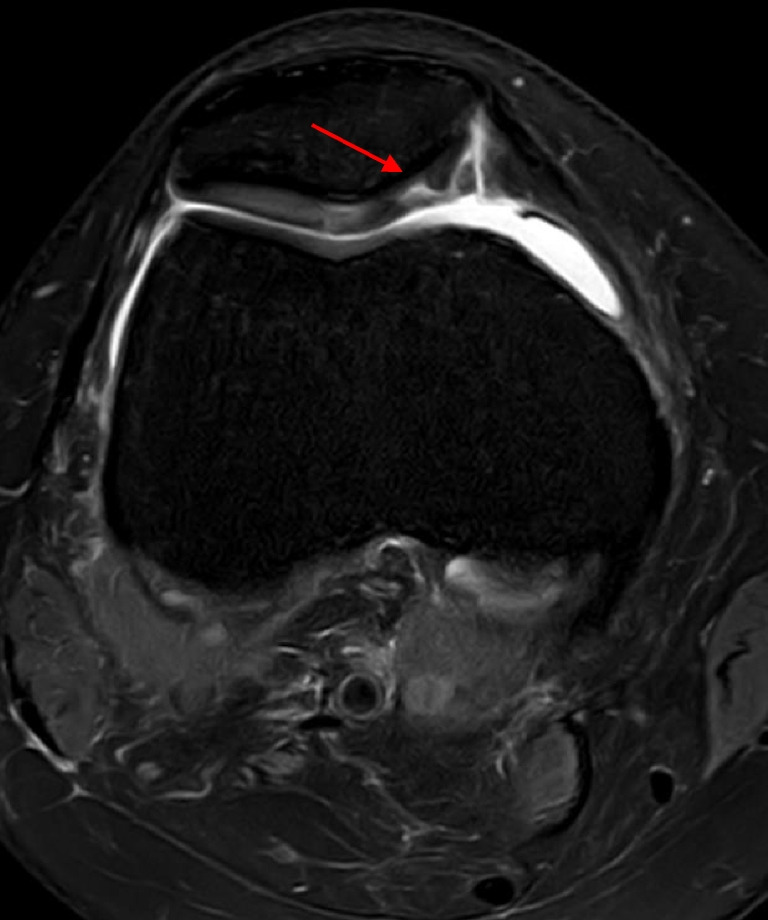
Full-thickness cartilage tear on the medial facet of the patella (arrow) with delamination of the adjacent cartilage on axial proton density fat saturation magnetic resonance imaging sequence (right knee)

Cartilage injuries were identified in 38 (15.0%) patients. Partial- and full-thickness tears (Fig. 7) and cartilage degeneration were observed in 25 (65.8%), nine (23.7%), and four (10.5%) patients, respectively. The injury locations were as follows: MFC (five patients, 13.2%), LFC (13 patients, 34.2%), lateral tibial plateau (two patients, 5.3%), trochlea (one patient, 2.6%), and patella (17 patients, 44.7%).

### Bone bruising, impaction fractures, and Segond fracture

**Fig. 8:**
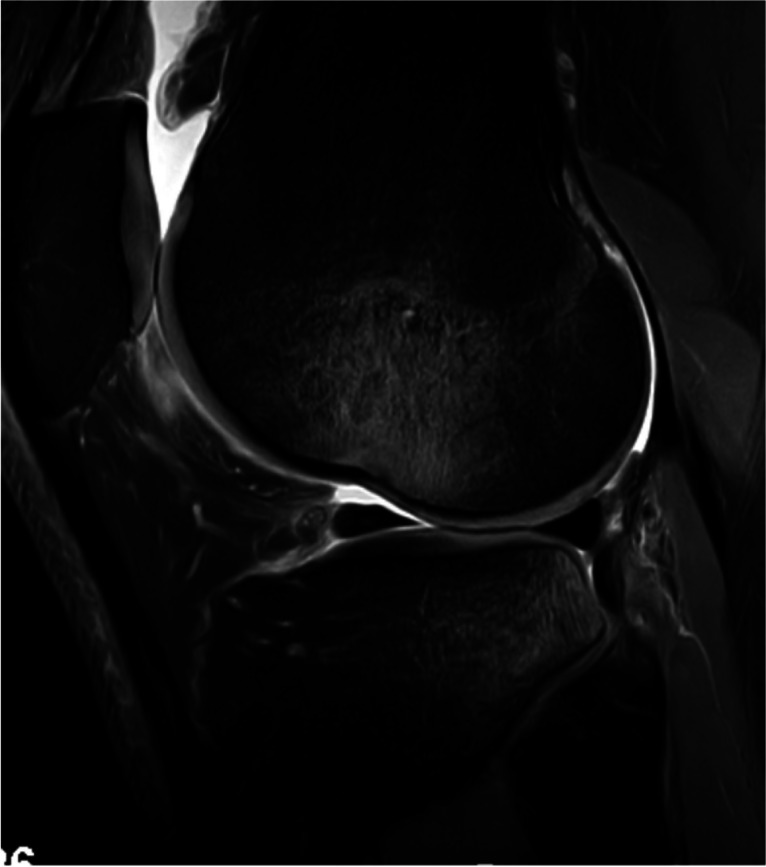
Pivot-shift type bone bruising as shown by the presence of bone marrow edema in the posterolateral area of the lateral tibial plateau and the central area of the lateral femoral condyle on sagittal proton density fat saturation magnetic resonance imaging sequence (left knee)

**Fig. 9:**
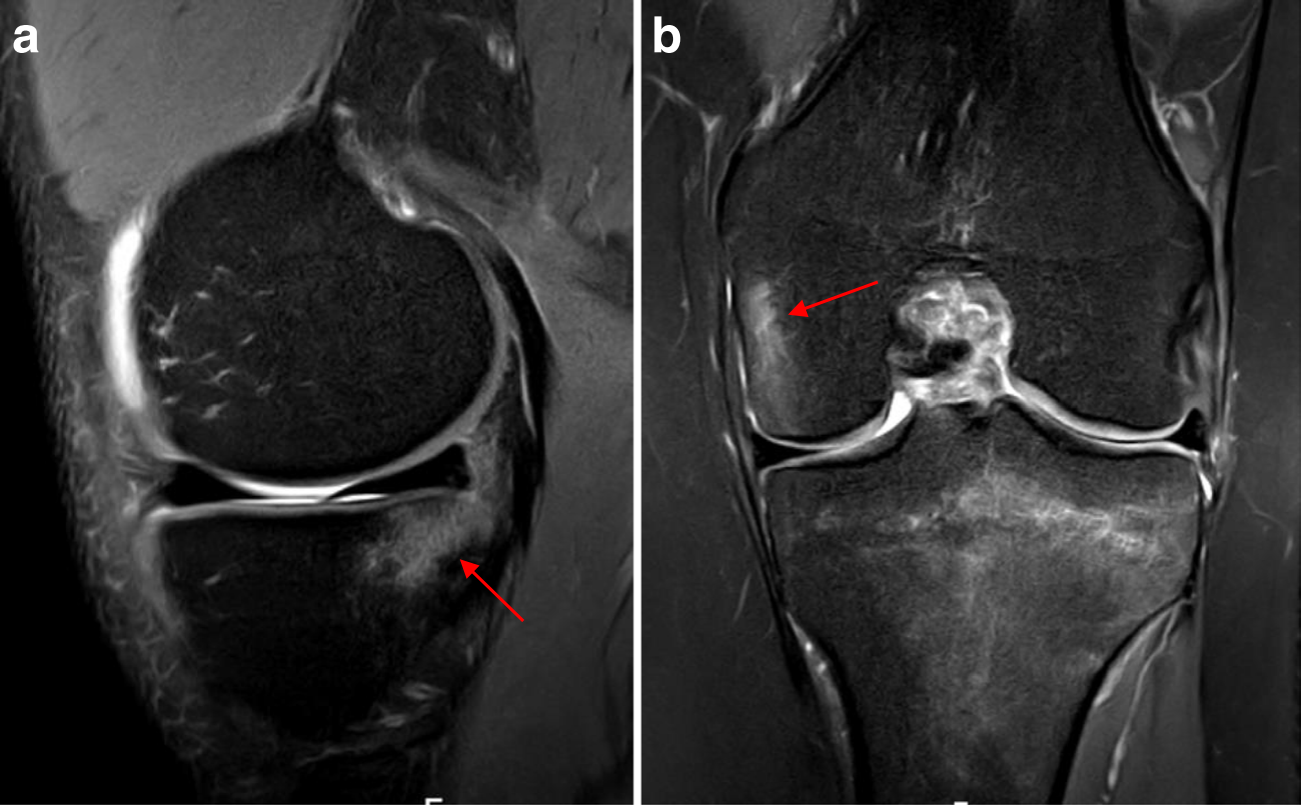
Posteromedial tibial (a) and medial femoral condyle (b) bone bruising (arrows) on sagittal and coronal proton density fat saturation magnetic resonance imaging sequence (left knee)

Bone bruising was observed in 236 patients (92.9%). The injury patterns were as follows: pivot shift (Fig. 8) (184 patients, 78%) and other (52 patients, 22%). Dashboard or hyperextension patterns were not observed. Additionally, MFC and PMT bone bruising (Fig. 9) were observed in 48 (18.9%) and 100 (39.4%) patients, respectively.

**Fig. 10:**
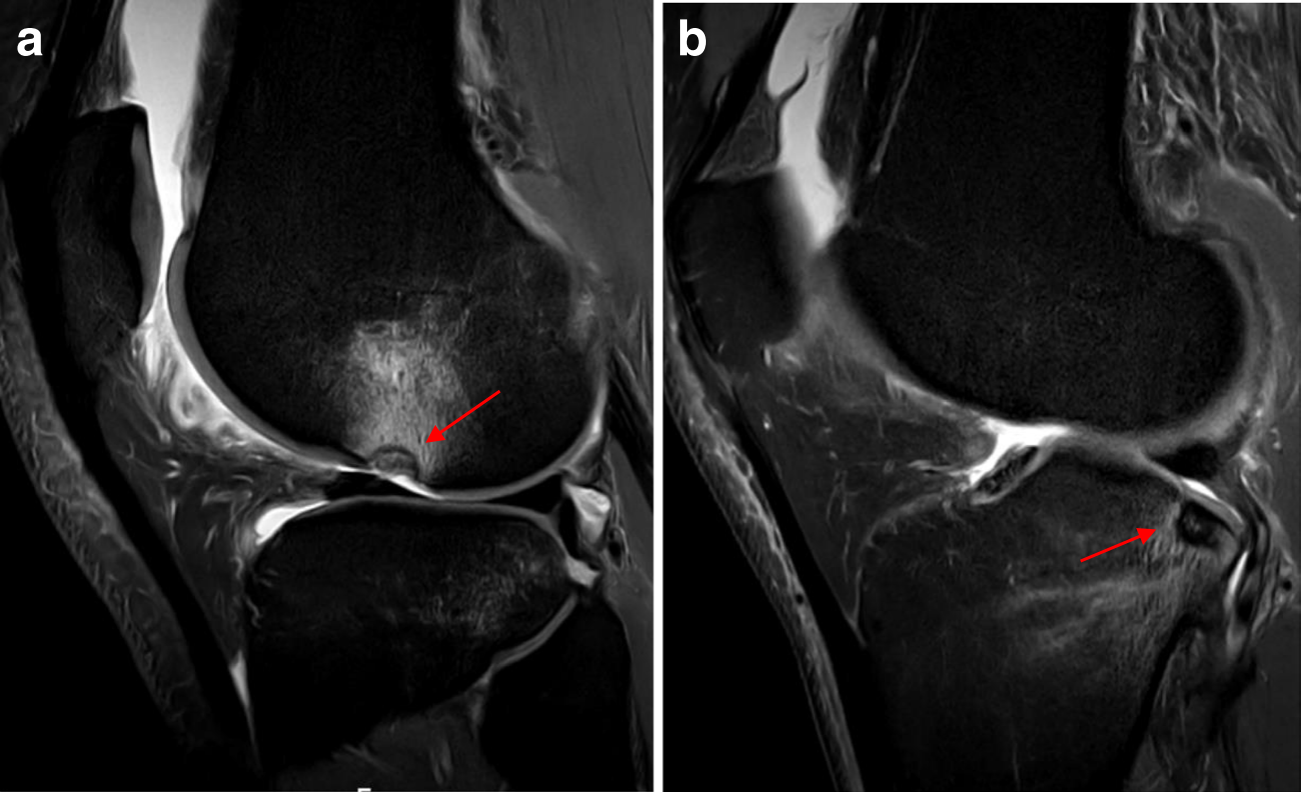
Impaction fracture in the lateral femoral condyle (a) and posterolateral tibia (b) (arrows) on sagittal proton density fat saturation magnetic resonance imaging (right knee)

**Fig. 11:**
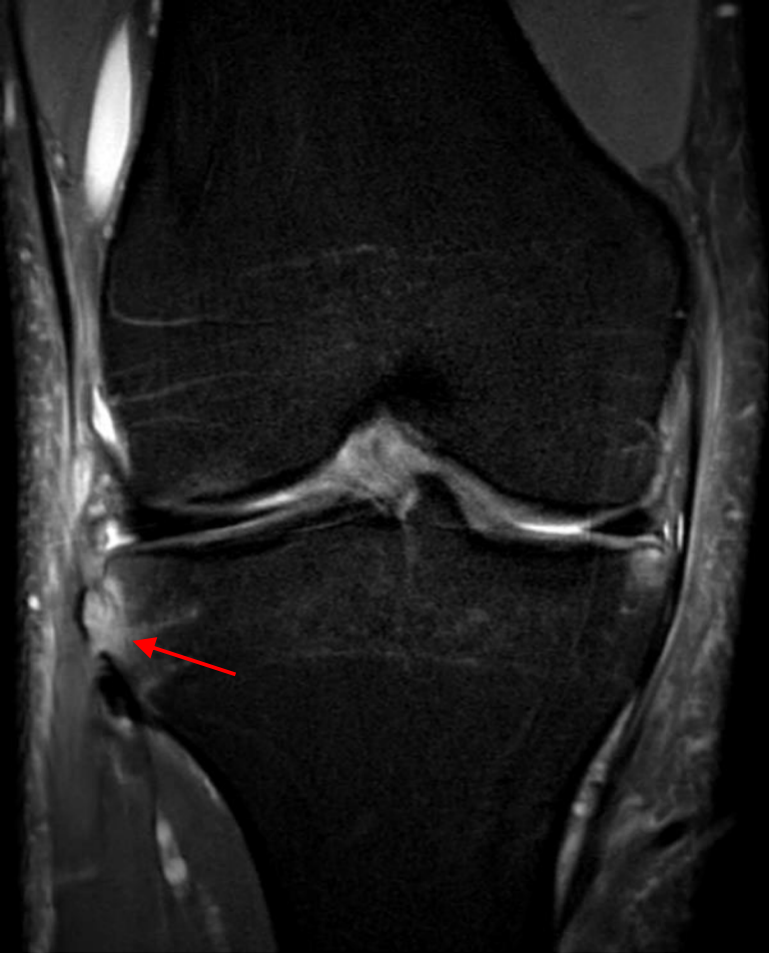
Segond fracture (arrow) on coronal proton density fat saturation magnetic resonance imaging (right knee)

Impaction fractures of the LFC and PLT (Fig. 10) were identified in 116 (45.7%) and 12 (4.7%) patients, respectively. Nineteen (7.5%) patients had Segond fractures (Fig. 11).

## Discussion

The most important finding of this study was the high prevalence of associated injuries in patients with ACL tears. This is consistent with previous research, suggesting that ACL tears are rarely isolated [1–4, 9, 21].

In the present study, the prevalence of MCL (sMCL and dMCL) and isolated dMCL injuries was 41.3% (16.5% + 24.8%). POL injuries were identified in 4.7% of patients and were never isolated. The aforementioned injuries were always associated with MCL (sMCL and dMCL) tears. Previous MRI studies have reported a variable prevalence (22–67%) of MCL complex injuries [4, 31–33]. This large variation may be the result of different intervals from injury to MRI as well as differences in the assessment and criteria used to define injuries to the MCL complex structures (sMCL, dMCL, and POL). In the present study, partial or complete sMCL tears were always associated with dMCL (meniscofemoral ligament) tears [2]. The load to failure of the dMCL is significantly lower than that of the sMCL (101 N and 557 N, respectively) [34]. A dMCL tear may occur before an sMCL tear. This may also explain why dMCL tears occurred in isolation (with intact sMCL). In the event of isolated dMCL tears, the meniscofemoral ligament was injured in all but one case. This ligament is thinner than the meniscotibial ligament [35, 36].

Lateral collateral ligament injuries were rare (2.4%). This might be related to the fact that the most common mechanism of ACL injuries in sports activities is a valgus-external rotation (“knee-in & toe-out”) [37], whereas the LCL is the primary restraint to varus loading [38]. The same consideration could be applied to popliteus tendon injuries which were not identified in any patient of the present cohort. However, recognition of these injuries is important, as a deficiency in the posterolateral corner (PLC) structures is responsible for persistent laxity and increased loads on the ACL graft [6, 39]. A previous study reported high rates (50–76%) of missed clinical diagnoses of PLC injuries by orthopedic surgeons [40].

Overall, MM and LM injuries were the most common (57.4% and 25.2%, respectively). Comparisons of prevalence with other studies are difficult as most of the relevant literature is based on arthroscopic findings [3, 9, 11, 21, 41]. Most meniscal injuries occurred in the posterior horn of the meniscus. This is not surprising because the posterior horns of the MM and LM provide secondary stability against anterior tibial translation [42] and internal tibial rotation [43], respectively. Interestingly, the most common meniscal injuries were MM ramp lesions. These injuries were present in 39.4% of the patients, as previously reported (overall 68.5% of MM injuries) [1]. Even though arthroscopy is generally regarded as the gold standard for the diagnosis of ramp lesions, previous studies have reported a significantly low prevalence (9–17%) of meniscal ramp lesions diagnosed arthroscopically at the time of ACLR [44–47]. This may be because the standard anteromedial and anterolateral portals have low sensitivity when diagnosing ramp lesions [14]. Sonnery-Cottet et al. [14] reported a 40% prevalence of meniscal ramp lesions (similar to that in our study) during ACLR. Interestingly, only 58% of these tears were diagnosed using the modified Gillquist view. Forty-two percent were diagnosed after probing and debridement through the posteromedial portal. Previous studies may have overlooked a significant number of meniscal ramp lesions identified by Sonnery-Cottet et al. [14] after debridement through a posteromedial portal and by us via MRI. Similar to our study, Balazs et al. [48] reported an overall MRI prevalence of meniscal ramp lesions of 42% in patients with ACL injuries. The meniscal ramp area can be visualized using an MRI performed with an appropriate magnetic field strength and spatial resolution [48].

Cartilage injuries were identified in 15% of the patients. Previous studies have reported a higher prevalence (16–28%) of cartilage injuries diagnosed arthroscopically at the time of ACLR [3, 9, 11, 49]. Compared with arthroscopy, MRI has moderate sensitivity for detecting cartilage injuries [50]. In addition, the risk of cartilage injury is directly correlated with the increased time since ACL injury [3, 9]. In the present study, the mean time from injury to MRI was relatively short (19.6 ± 15.2 days). These factors may explain the lower prevalence of cartilage injuries in this study than that in previous studies.

Bone bruising was observed in most patients (92.9%). The most frequent pattern (78% of patients) was the pivot-shift pattern (bone marrow edema in the posterolateral area of the lateral tibial plateau and central area of the LFC). This might be explained by the fact that most ACL injuries and the pivot-shift bone-bruising pattern share the same trauma mechanism [flexion valgus and external rotation (pivot-shift)] [19, 51, 52]. It is hypothesized that, during the pivot shift mechanism, the posterolateral aspect of the lateral tibial plateau subluxates anteriorly and impinges with the central part of the LFC [19, 21]. In line with the present study, Yoon et al. [21] reported a high prevalence (84%) of bone bruising in patients with ACL injuries (MRI performed within 6 weeks of the trauma), with most bone bruising occurring in the LFC (68%) and the lateral tibial plateau (73%). These findings support the hypothesis that bone bruising should be interpreted as a footprint of the mechanism of injury [19]. Impaction fractures in the LFC and PLT (present in 45.7% and 4.7% of the patients, respectively) can also be observed in the context of a pivot-shift mechanism [19, 53]. Interestingly, these impaction fractures are associated with progressive cartilage degeneration, greater anterolateral rotatory laxity, and poorer postoperative outcomes [23, 24]. Bone bruising was also commonly observed in the MFC and PMT (18.9% and 39.4%, respectively). It has been hypothesized that bone bruising in the medial compartment occurs as the result of a contrecoup mechanism. If energy trauma is not dissipated by the initial pivot-shift mechanism, a contrecoup injury with impaction of the MFC and PMT may occur as a result of sudden tibial reduction with compensatory varus alignment and internal tibial rotation [52]. However, we previously reported a strong association between MFC bone bruising and dMCL injuries in the same patient cohort from the NACOX study [2]. We hypothesized that the avulsion of the meniscofemoral ligament from the MFC may be responsible for bone bruising in the MFC. PMT bone bruising has been strongly associated with MM ramp lesions [1] as they probably share the same trauma mechanism [1, 54].

Segond fractures were present in a small number of patients (7.5%). This rate is consistent with that reported in the literature [55]. The low rate of Segond fractures may be explained by the fact that this injury is thought to occur after internal rotation and varus stress [30], whereas most ACL injuries occur after external rotation and valgus stress [37]. However, it is important to note that this injury has been associated with increased anterolateral rotatory laxity in ACL-injured knee [25].

MRI is generally considered the best imaging modality for diagnosing associated injuries in patients with ACL tears. Although MRI cannot be regarded as a substitute for a thorough clinical examination, it can serve as an alert to orthopedic surgeons, guiding them to pay particular attention (during the clinical assessment) to the injured structures on imaging [2]. Additionally, MRI is useful for preoperative planning and can affect clinical decision-making in patients with ACL injuries.

The main strength of this study was that all MRI scans were reviewed by two experienced examiners (an orthopedic surgeon specializing in knee surgery and a musculoskeletal radiologist). A relatively high prevalence (54.5%) of MRI scans was obtained using a 3.0 Tesla scanner. Moreover, the time from injury to MRI was relatively short (19.6 ± 15.2 days). This prevented the edema in the injured structures (and bone bruising) from resolving and improved the diagnosis of the associated injuries. Associated injuries were evaluated and reported according to established classifications [19, 28]. Furthermore, meniscal ramp lesions were evaluated using well-defined MRI pathological signs [29]. The injury patterns of the MCL complex (sMCL, dMCL, and POL) were analyzed in detail. This is clinically relevant because, in the event of surgical treatment, all MCL complex structures should be repaired or reconstructed [56]. Finally, the cohort studied (n = 254) was relatively large.

Some limitations are present. First, the patients underwent MRI scans at different institutions using different scanners. However, all examinations were performed with a strength of 1.5 or 3.0 T. Moreover, the MRIs were assessed by the same two examiners. Second, some associated injuries (ex. cartilage injuries in the patellofemoral joint) are unlikely to have occurred in concomitance with the ACL injury. However, the purpose of the study was to provide an overview of all associated injuries in the ACL-injured patient, rather than evaluating only them possibly occurring in concomitance with the ACL tear.

In conclusion, the prevalence of associated injuries in patients with ACL tears was very high. The findings reported in this study may serve as a reference tool for orthopedic surgeons and radiologists in the diagnosis of associated injuries in patients with ACL tears using MRI.

## Appendix

**Table 4:** Sensitivity analysis of MRI scanners

	1.5-T MRI (*n* = 115)	3.0-T MRI (*n* = 139)	*P* Value^a^
MCL injury	18 (15.5)	24 (17.7)	0.73
Partial rupture	12 (10.3)	17 (12.3)	0.20
Complete disruption	6 (5.2)	7 (5.1)	
Isolated deep MCL injury	25 (21.6)	38 (27.5)	0.30
LCL injury (partial rupture)	3 (2.6)	3 (2.2)	0.83
MM injury	63 (54.3)	83 (60.6)	0.31
Ramp lesions	44 (37.9)	56 (40.9)	0.63
LM injury	18 (15.5)	46 (33.3)	0.001*
Cartilage injury	14 (12.1)	20 (14.5)	0.57
Partial thickness	12 (10.3)	13 (9.4)	0.13
Full thickness	2 (1.7)	7 (5.1)	
Degeneration	0 (0.0)	4 (2.9)	
BML	107 (93.0)	129 (92.8)	0.32
Pivot-shift type	88 (75.9)	96 (69.6)	
Other	19 (16.4)	33 (23.9)	
BML in the PMT	38 (32.8)	62 (44.9)	0.05
BML in the MFC	7 (6.0)	41 (29.7)	< 0.001*
Impaction fracture of the LFC	47 (40.5)	69 (50.0)	0.13
Segond fracture	7 (6.0)	12 (8.7)	0.42

Data are reported as number (percentage)

*BML* bone-marrow lesion, *LCL* lateral collateral ligament, *LFC* lateral femoral condyle, *LM* lateral meniscus, *MCL* medial collateral ligament, *MFC* medial femoral condyle, *MM* medial meniscus, *MRI* magnetic resonance imaging, *PMT* posteromedial tibia

^a^ Pearson chi-square test or Fisher´s exact test

*Statistically significant
